# Supplementary material for: Constraining the trend in the ocean CO2 sink during 2000–2022
Source: Nat Commun. 2024 Sep 28;15:8429. doi: 10.1038/s41467-024-52641-7 (PMC11438992; doi:10.1038/s41467-024-52641-7)
Supplement: Supplementary file 1 — Supplementary Information [file 41467_2024_52641_MOESM1_ESM.pdf]

Constraining the trend in the ocean CO_2 sink during 2000-2022

Nicolas Mayot[1*]
Erik T. Buitenhuis[1]
Rebecca M. Wright[1]
Judith Hauck[2]
Dorothee C. E. Bakker[1]
Corinne Le Quéré[1]

Supplementary Information

[1]School of Environmental Sciences, University of East Anglia, Norwich, UK, [2]Alfred-Wegener-Institut, Helmholtz-Zentrum für Polar- und Meeresforschung, Bremerhaven, Germany.*e-mail: n.mayot@uea.ac.uk

Supplementary Table 1 | Values of RMSE, and of interannual (Int.) and decadal (Dec.) variability from the ensemble estimates used within the global carbon budget analysis in 2023, the PlankTOM12.1 simulations and the hybrid approach. The metrics were calculated globally and for three latitudinal bands over the period 1990-2022. RMSE is in µatm, and interannual and decadal variability values are in Pg C yr^{-1}. For the fCO_2-products, the GOBMs, and the perturbed simulations, the value in bold is the average, and the values above and below indicate the range (minimum and maximum). For the hybrid approach, results for uncertain years were discarded.

**Product names**	**Global**			**North**			**Tropics**			**South**		
	**RMSE**	**Int.**	**Dec.**	**RMSE**	**Int.**	**Dec.**	**RMSE**	**Int.**	**Dec.**	**RMSE**	**Int.**	**Dec.**
GCB's fCO_2-products	14.3	0.07	0.06	18.0	0.02	0.005	8.9	0.03	0.01	14.0	0.02	0.001
	**20.3**	**0.11**	**0.14**	**22.7**	**0.03**	**0.01**	**16.7**	**0.06**	**0.02**	**20.9**	**0.05**	**0.01**
	26.3	0.23	0.21	29.9	0.07	0.01	21.0	0.09	0.03	27.4	0.10	0.03
GCB's GOBMs	31.3	0.07	0.06	37.2	0.01	0.002	22.7	0.03	0.01	32.3	0.02	0.001
	**39.0**	**0.11**	**0.08**	**46.0**	**0.02**	**0.008**	**28.9**	**0.07**	**0.02**	**38.1**	**0.04**	**0.01**
	45.0	0.15	0.11	53.5	0.06	0.015	34.7	0.10	0.04	45.6	0.07	0.02
NEMO-PlankTOM12.1	38.5	0.10	0.11	45.8	0.01	0.004	24.4	0.06	0.04	44.0	0.03	0.01
Perturbed simulations	38.6	0.10	0.05	45.9	0.02	0.02	23.8	0.07	0.03	43.0	0.03	0.02
	**39.9**	**0.10**	**0.10**	**47.0**	**0.02**	**0.02**	**26.3**	**0.08**	**0.07**	**45.4**	**0.04**	**0.03**
	41.4	0.12	0.12	49.1	0.03	0.02	29.5	0.10	0.10	49.9	0.04	0.03
Hybrid approach	38.0	0.22	0.13	45.6	0.03	0.004	23.2	0.08	0.02	42.5	0.06	0.01

Supplementary Table 2 | Regional decadal trend values from the ensemble estimates used within the global carbon budget analysis in 2022, the PlankTOM12.1 simulations and the hybrid approach. The trends are calculated over three periods: 2000s, 2010s and 2000-2022. All values are in Pg C yr^{-1} $decade^{-1}$.

**Product names**	**North**			**Tropics**			**South**		
	**2000s**	**2010s**	**2000 - 2022**	**2000s**	**2010s**	**2000 - 2022**	**2000s**	**2010s**	**2000 - 2022**
GCB's fCO_2-products	0.20 ± 0.14	0.12 ± 0.04	0.12 ± 0.02	0.20 ± 0.11	0.32 ± 0.11	0.13 ± 0.04	0.29 ± 0.24	0.21 ± 0.14	0.28 ± 0.10
GCB's GOBMs	0.09 ± 0.03	0.05 ± 0.06	0.05 ± 0.02	0.18 ± 0.10	0.14 ± 0.09	0.10 ± 0.03	0.17 ± 0.04	0.13 ± 0.07	0.13 ± 0.04
NEMO-PlankTOM12.1	0.10	0.02	0.05	-0.002	0.24	0.10	0.15	0.22	0.17
Hybrid approach	0.33 ± 0.06	–0.02 ± 0.06	0.09 ± 0.02	0.18 ± 0.02	0.06 ± 0.04	0.12 ± 0.01	0.14 ± 0.25	0.51 ± 0.18	0.27 ± 0.07

Supplementary Table 3 | Temporal variability of the ocean CO_2 sink estimated using the different set ups on which the hybrid approach was applied. The six different set ups are shown in Supplementary Figure 5, and the associated global ocean CO_2 sink estimates in Supplementary Figure 6. The first row represents the set up presented in the main manuscript. The last row represents the ensemble average (± 1σ) of all set ups. The last column shows the number of years, between 1990 and 2022, with a constrained estimate of the annual ocean CO_2 sink for each configuration.

	**Variability amplitude (1990-2022, in Pg C yr^{-1})**		**Decadal trends (Pg C yr^{-1} decade^{-1})**				**Number of constrained years (uncertain years)**
	**Interannual**	**Decadal**	**1990s**	**2000s**	**2010s**	**2000-2022**	
NCEP and bact.	0.22	0.13	-0.19	0.80	0.44	0.42	32 (0)
ERA5 and bact.	0.23	0.25	-0.93	1.27	0.35	0.36	33 (0)
NCEP and phyto.	0.28	0.16	0.17	0.97	0.48	0.27	25 (7)
ERA5 and phyto.	0.19	0.18	-0.60	0.87	0.23	0.24	32 (8)
NCEP and bact. & phyto.	0.18	0.09	-0.37	0.73	0.37	0.41	27 (0)
ERA5 and bact. & phyto.	0.17	0.14	-0.43	1.14	0.23	0.36	31 (2)
Mean ± 1σ	0.21 ± 0.04	0.16 ± 0.05	-0.43 ± 0.37	0.96 ± 0.21	0.35 ± 0.10	0.34 ± 0.07	

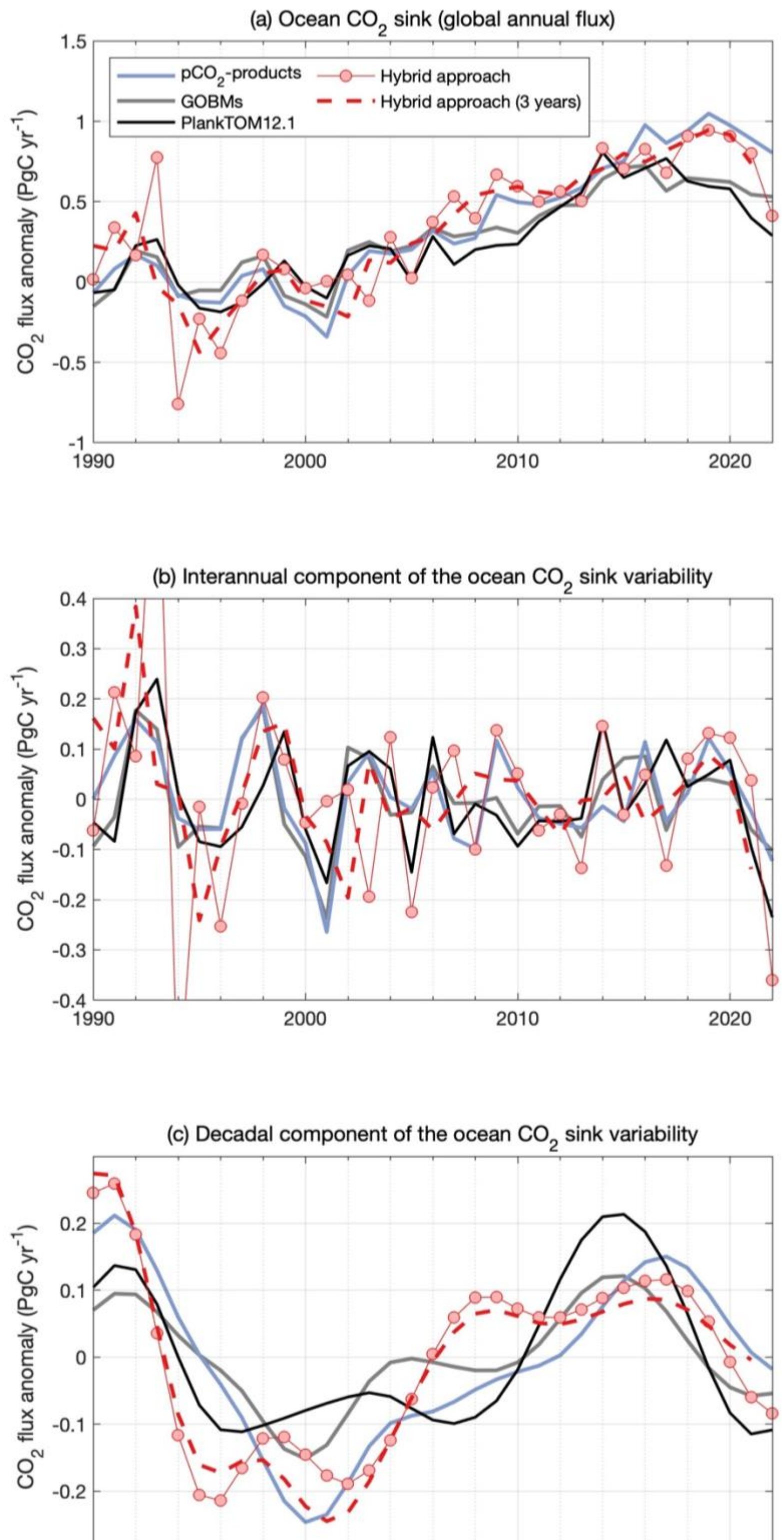

Supplementary Fig. S1 | Temporal variations in the various estimates of the ocean CO_2 sink. a Global annual flux from the GOBMs (grey), the fCO_2-products (blue), NEMO-PlankTOM12.1 (black), the hybrid approach (red) and the hybrid approach performed with three consecutive years (dashed red). The variability of the global annual flux has been decomposed into **b** an interannual component and **c** a decadal component.

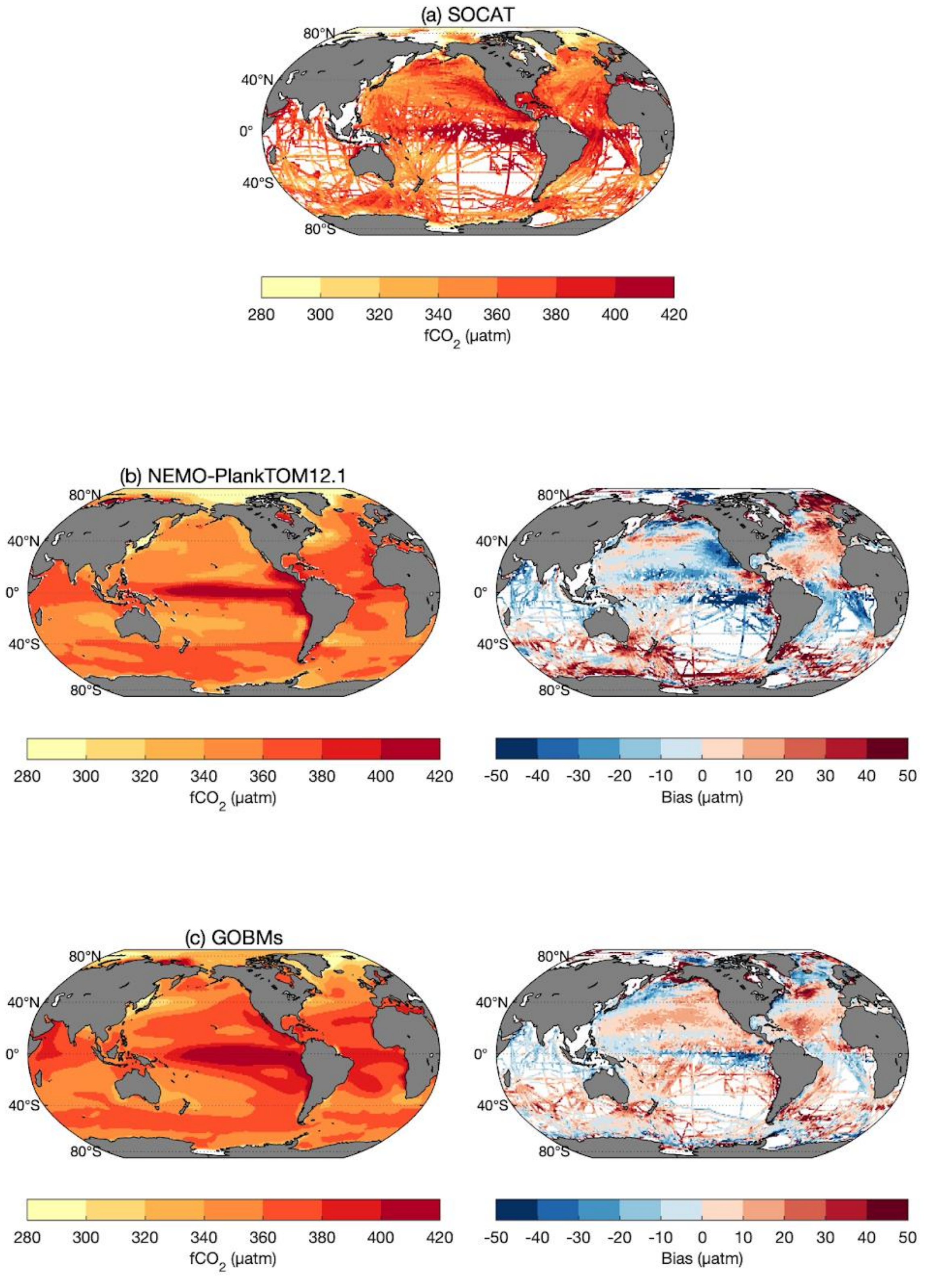

Supplementary Fig. S2 | Spatial distribution of mean bias between SOCAT and: NEMO-PlankTOM12.1 and the GOBMs from the global carbon budget analysis 2023, calculated over the period 1990-2022. a Surface fCO_2 observations from SOCAT averaged at each location. Note that most locations are not sampled every year. **b-c** The mean surface fCO_2 and bias between SOCAT and: **b** NEMO-PlankTOM12.1, **c** the GOBMs. For the GOBMs, a map is calculated for each GOBM, and the average map is displayed. Note that NEMO-PlankTOM12.1 is removed from the GOBM ensemble.

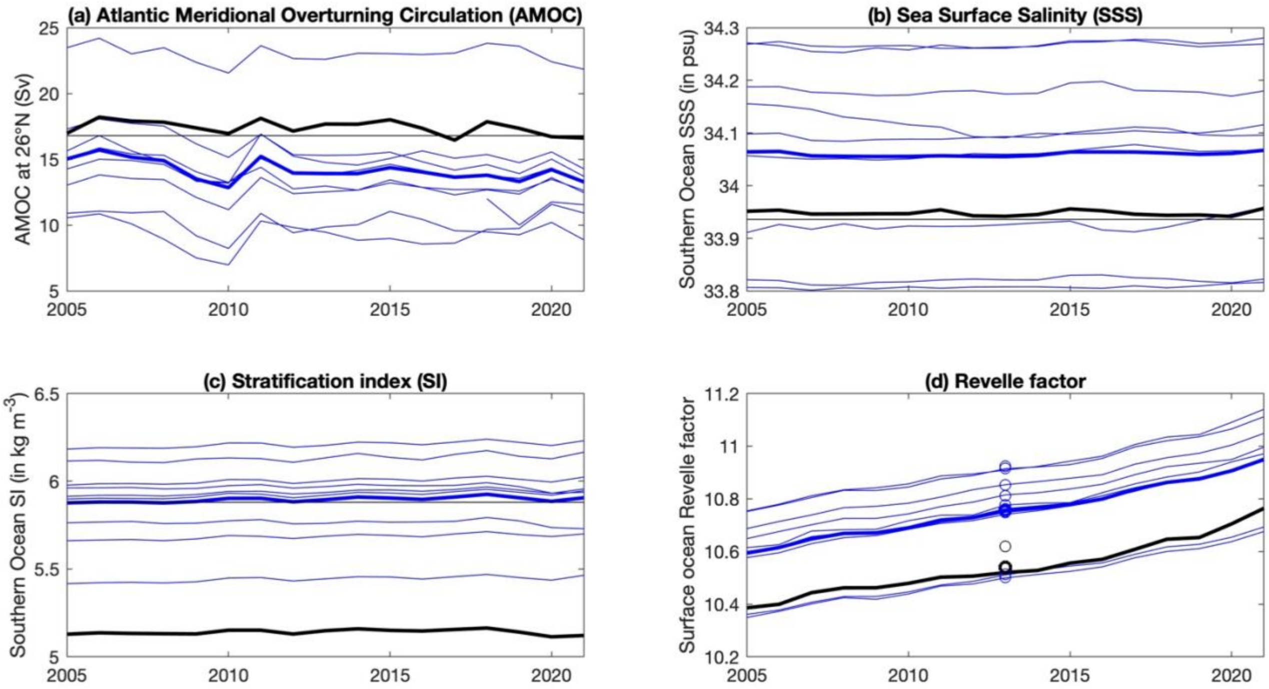

Supplementary Fig. S3 | Validation of NEMO-PlankTOM12.1 and GOBMs from the global carbon budget analysis in 2023. The variables used for the validation are **a** the Atlantic Meridional Overturning Circulation at 26°N, **b** the Southern Ocean sea surface salinity (SSS), **c** the Southern Ocean Stratification Index (SI), and **d** the surface ocean Revelle factor (global average). The individual estimate from GOBMs (thin blue lines) are averaged (thick blue line). The observed estimate averaged over the period 2005-2021 is the thin black line. The NEMO-PlankTOM12.1 estimate is the thick black line. For the Revelle factor, the circles correspond to the 2005-2021 average. The observation-based AMOC is from the RAPID-MOCHA-WBTS array. The observed SSS and SI are estimated from the temperature and salinity data of the EN4 product. The observation-based Revelle factor value is from the OceanSODA fCO_2-product.

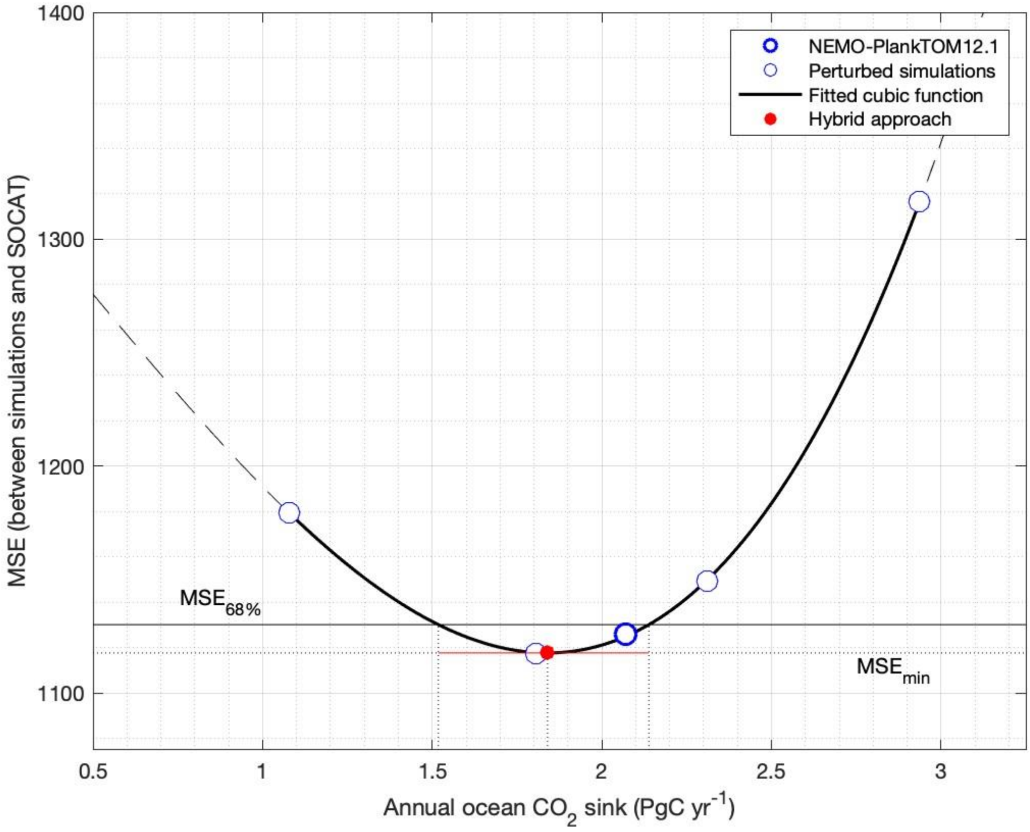

Supplementary Fig. S4 | Graphical interpretation of the hybrid approach. In this example, the values for the year 2002 are represented. The hybrid approach requires four perturbed simulations of NEMO-PlankTOM12.1 (blue circles). For each simulation, and for the year 2002, the MSE value is shown on the *y*-axis and the ocean CO_2 sink estimate on the *x*-axis. A cubic function is fitted to these data points (thick black line). The constrained ocean CO_2 sink for the year 2002 is estimated by finding the local minimum (inflection point) associated with the rising concave section of the fitted cubic function.

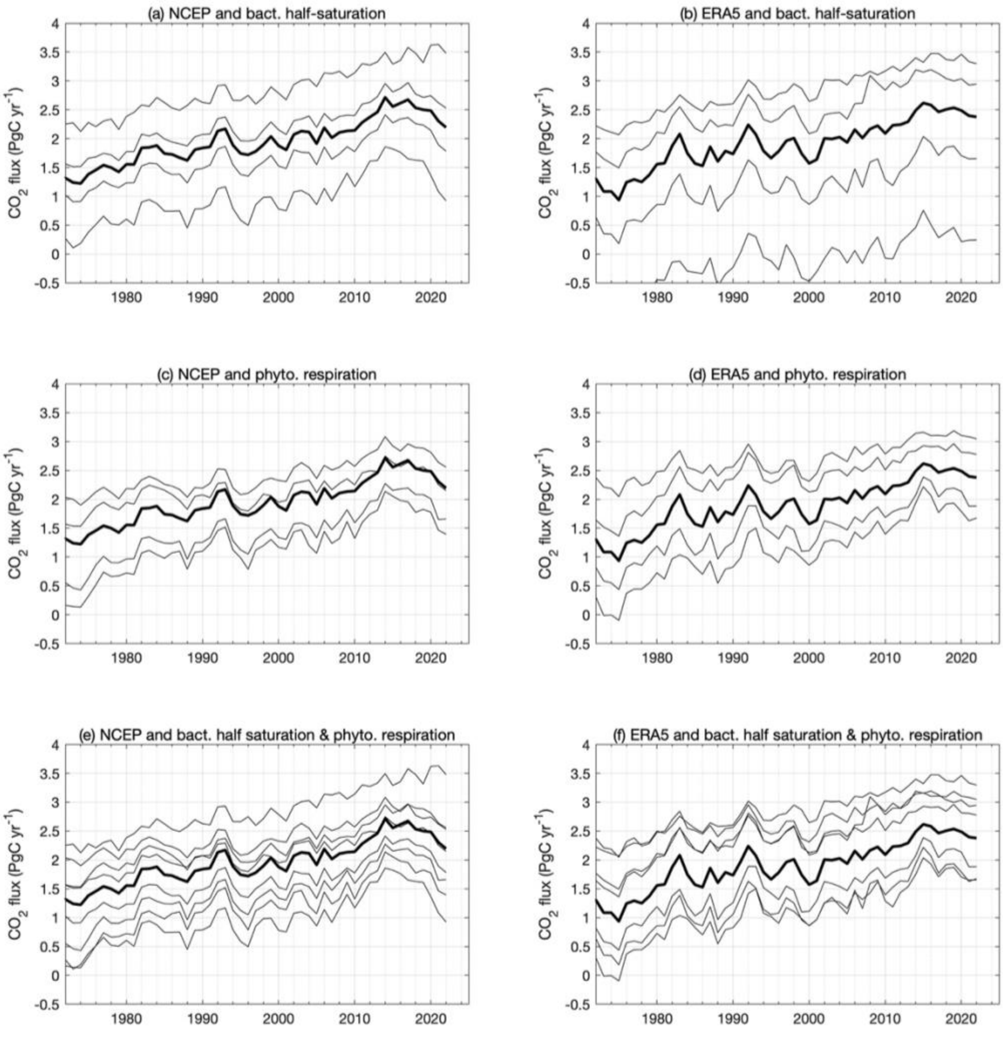

Supplementary Fig. S5 | Ocean CO_2 sink, for the global ocean, from the six different set ups on which the hybrid approach was applied. The set ups are **a** NCEP reanalysis with bacterial half-saturation, **b** ERA5 reanalysis with bacterial half-saturation, **c** NCEP reanalysis with phytoplankton respiration, **d** ERA5 reanalysis with phytoplankton respiration, **e** NCEP reanalysis with bacterial half-saturation and phytoplankton respiration, and **f** ERA5 reanalysis with bacterial half-saturation and phytoplankton respiration. The thick line is the standard simulation with NCEP or ERA5, and the thin lines are the perturbed simulations. In the main manuscript we show results obtained when applying the hybrid approach with the model forced with NCEP and the perturbation of the half-saturation constant of bacterial remineralisation (**a**).

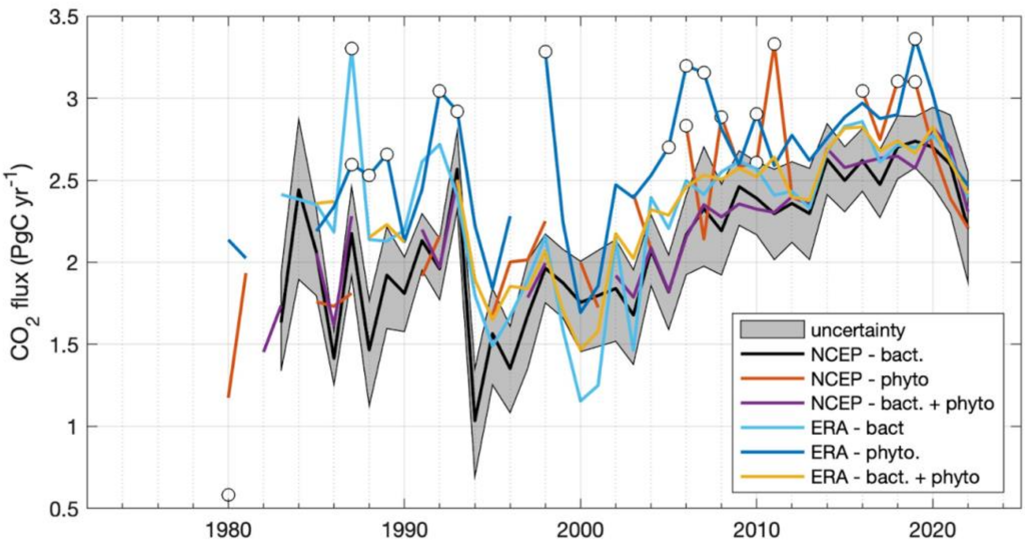

Supplementary Fig. S6 | Hybrid approach results when using different perturbed parameters. The black line with the grey shading, represents the results and error estimate from the main manuscript (using NEMO-PlankTOM12.1 forced with NCEP, and perturbed bacteria). The other coloured lines represent hybrid approach results obtained with a different forcing (i.e., ERA), and/or when perturbing phytoplankton and/or bacteria. The white dots represent uncertain results as defined in the manuscript.

Equations of the global carbon budget analysis:

Within the global carbon budget analysis[1], the carbon sinks (atmosphere = G_{ATM}, ocean = S_{OCEAN}, and land = S_{LAND}) and emissions (from fossil fuel = E_{FOS}, and land-use changes = E_{LUC}) are estimated,

$$(G_{ATM} + S_{OCEAN} + S_{LAND}) = E_{FOS} + E_{LUC} \qquad (1)$$

By using the estimates of G_{ATM}, E_{FOS} (including cement carbonation), and E_{LUC} from the Global Carbon Budget analysis published in 2023, with our estimate of S_{OCEAN} from the hybrid approach, an estimate of the total land CO_2 sink can be obtained:

$$\text{Total land } CO_2 \text{ sink} = (S_{LAND} - E_{LUC}) = E_{FOS} - (G_{ATM} + S_{OCEAN}) \qquad (2)$$

Note that the difference between the terms in equation 1 is equal to the Budget Imbalance (B_{IM}) of the global carbon budget:

$$BIM = E_{FOS} + E_{LUC} - (G_{ATM} + S_{OCEAN} + S_{LAND}) \qquad (3)$$

Constraints on the interannual variability of the annual global ocean CO_2 sink:

An estimation of the interannual variability of the global ocean CO_2 sink can be obtained by removing the decadal component from the original detrended time series of the annual ocean CO_2 sink (Supplementary Fig. S1b). The hybrid approach preserves the patterns of interannual variability from the NEMO-PlankTOM12.1 (Supplementary Fig. S1b; $r = 0.5$, $p = 0.004$, Pearson's correlation coefficient), but double its magnitude to 0.22 Pg C yr^{-1}. Originally, over the period 1990-2022, NEMO-PlankTOM12.1 simulated amplitudes of interannual variability for the ocean CO_2 sink (0.10 Pg C yr^{-1}) comparable to the interannual variability simulated by the other GOBMs and fCO_2-products used in the global carbon budget analysis (0.11 ± 0.02 Pg C yr^{-1} and 0.11 ± 0.06 Pg C yr^{-1}, respectively). Note that the hybrid approach also increased the regional interannual variability (Supplementary Table 2).

As for the decadal variability, we tested the robustness of this interannual variability estimate with respect to (i) the choice in the selected model's configuration and parameter perturbed, and (ii) the annual availability and distribution of SOCAT data. The interannual variability from the six different model set ups used (see methods, section 5.3 for more details) were comparable (0.21 ± 0.04 Pg C yr^{-1}). However, when the hybrid approach was applied by considering observations from three consecutive years, the interannual variability was strongly reduced to 0.11 ± 0.01 Pg C yr^{-1} (Supplementary Fig. S1b). This reduction of the interannual variability was mostly observed in the 1990s, when fewer observations were available. This 3-year interannual variability value was still larger than that estimated by GOBMs (0.06 ± 0.01 Pg C yr^{-1}), NEMO-PlankTOM12.1 (0.06 Pg C yr^{-1}), and fCO_2-products (0.07 ± 0.03 Pg C yr^{-1}), when smoothed with a 3-year running mean. Overall, results from the sensitivity analyses suggest that a significant uncertainty surrounding the amplitude of interannual variability remains.

Nonetheless, despite remaining uncertainty on its amplitude, our results confirm the general consensus for the temporal patterns of interannual variability, common among the various approaches, in agreement with other studies[2,3]. The fact that the hybrid approach preserves the patterns of interannual variability from the NEMO-PlankTOM12.1, but increased its

magnitude, could suggest that NEMO-PlankTOM12.1 and other GOBMs represent the correct processes, but either they do not respond sufficiently to changes in external forcing, or the balance among thermal and non-thermal processes in response to external forcing is imperfect[4]. For example, in the Southern Ocean, ocean surface fCO_2 variations over the year in NEMO-PlankTOM12.1, and in most GOBMs, tend to be too strongly influenced by temperature changes[5]. Additional fCO_2 sampling, mostly at high latitudes, could help constrain the amplitude of the interannual variability obtained by our hybrid approach and resolve some of the identified issues here and in the literature[5].

Supplementary References:

1. Friedlingstein, P. *et al.* Global Carbon Budget 2023. *Earth System Science Data* **15**, 5301–5369 (2023).
2. Bennington, V., Gloege, L. & McKinley, G. A. Variability in the Global Ocean Carbon Sink From 1959 to 2020 by Correcting Models With Observations. *Geophysical Research Letters* **49**, e2022GL098632 (2022).
3. Mayot, N. *et al.* Climate-driven variability of the Southern Ocean CO2 sink. *Philosophical Transactions of the Royal Society A: Mathematical, Physical and Engineering Sciences* **381**, 20220055 (2023).
4. Li, H., Ilyina, T., Müller, W. A. & Landschützer, P. Predicting the variable ocean carbon sink. *Science Advances* **5**, eaav6471 (2019).
5. Hauck, J. *et al.* The Southern Ocean Carbon Cycle 1985–2018: Mean, Seasonal Cycle, Trends, and Storage. *Global Biogeochemical Cycles* **37**, e2023GB007848 (2023).
